# Feasibility of Force-Sensing Finger Assessment in Elite Fencers: A Pilot Study with Clinical Translational Potential

**DOI:** 10.3390/jcm14207335

**Published:** 2025-10-17

**Authors:** Anna Akbaş, Michał Pawłowski

**Affiliations:** Department of Human Motor Behavior, Institute of Sport Sciences, Academy of Physical Education, 40-065 Katowice, Poland; m.pawlowski@awf.katowice.pl

**Keywords:** instrumented handle, force-sensitive resistor, fencing, multi-digit coordination, grip output, reliability, submaximal control, task-specific upper-limb functional output

## Abstract

**Background**: Grip control is a critical determinant of fencing performance, requiring both stability and precision. Traditional measures of hand strength, such as dynamometry, provide only a global estimate and cannot capture finger-specific load distribution. Yet, upper-extremity overuse syndromes, tendinopathies of the wrist and digital flexors are common in fencers, underscoring the need for more granular assessments that may inform clinical practice, especially in prehension contexts. **Methods**: This pilot study included eight elite épée fencers from the Polish National Team (age: 23.9 ± 4.9 years; training experience: >10 years) tested using a novel épée handle instrumented with five force-sensitive resistors (FSRs) embedded beneath each finger. Participants performed two 5-s maximal voluntary contractions (MVCs) for each of the three conditions—Pinch (thumb + index), Trio (middle + ring + small), and Whole (all digits). Standard handheld dynamometry was also performed to provide a global reference measure. **Results**: Maximal grip strength measured with a dynamometer (65.3 ± 11.7 kgf) was substantially higher than finger-specific forces captured with the FSR handle (14.4 ± 4.4 kgf). Isolated Pinch contractions (83.0 ± 29.2 N) were significantly stronger than their integrated contribution within the Whole-hand condition (54.7 ± 16.3 N; Z = 2.52, *p* = 0.012), whereas Trio forces did not differ significantly (*p* = 0.263). On average, radial digits (thumb + index) contributed ~39% and ulnar digits (middle, ring, small) ~61% of Whole output, with the thumb and middle finger producing the largest forces. **Conclusions**: This pilot study demonstrates the feasibility of using an FSR-instrumented épée handle to capture finger-specific grip contributions in elite fencers. Despite limited statistical power (n = 8), the observed effects provide initial quantitative evidence for sport-specific, digit-level assessment, showing potential clinical utility in detecting maladaptive load-transfer mechanisms and informing rehabilitation and injury-prevention programs.

## 1. Introduction

Fencing performance critically depends on the ability to control weapon grip with both stability and precision [1]. Unlike general measures of hand strength, fencing grips require finely tuned, finger-specific force application to stabilize the weapon while allowing rapid execution of technical actions [2]. Previous studies have demonstrated that handle design and material properties influence grip distribution, muscle activation, and even technical accuracy [3,4]. Yet, despite these insights, current assessment tools—such as dynamometers—provide only global information and cannot capture how individual fingers contribute to force generation during sport-specific tasks [5,6]. A growing body of research on multi-digit coordination confirms that the fine control of inter-finger forces and their dynamic synergies is a central determinant of skilled manual performance, supporting the need for finger-resolved measurement approaches [7].

From an injury-prevention perspective, this gap is particularly relevant. Although fencing is generally considered safe, upper-extremity injuries remain a consistent burden, reflecting the asymmetrical demands placed on the weapon arm [8,9]. In an extensive retrospective review, 16% of all fencing injuries involved the upper extremity, with the hand (35%), shoulder (31%), and wrist (24%) being the most affected sites [10]. Emergency department data further highlight the hand and fingers as common locations, accounting for 14% of acute injuries, often presenting as sprains, strains, or fractures. These injuries occur overwhelmingly on the dominant, weapon-holding side, where muscular hypertrophy and repetitive loading, although adaptive, may elevate localized tissue stress and reduce resilience to cumulative microtrauma, thereby increasing overuse risk. Equipment factors also modulate these demands: pistol handles have been shown to reduce activation of the adductor pollicis and extensor carpi radialis, potentially delaying neuromuscular fatigability while certain handle angles (18–21°) improve technical accuracy but may also increase susceptibility to localized overload [2,3]. Comparable observations have been made in other precision sports—such as tennis, golf, and climbing—where finger pressure distribution and grip asymmetries have been associated with performance efficiency and, in some cases, overuse-related loading of the wrist and forearm extensors [11,12,13,14]. These findings collectively point to the need for sport-specific tools that can quantify fine-grained load distribution at the finger level.

Despite extensive epidemiological data, there remains no direct method to quantify how finger-specific load-sharing contributes to either technical performance or the onset of overuse syndromes. Conventional dynamometry cannot discriminate between individual finger contributions, and EMG provides only indirect information on muscle activation rather than actual force transmission to the handle. This limits both the biomechanical understanding of grip control and its clinical application in injury prevention and rehabilitation. Beyond sport-specific considerations, grip strength has also been widely recognized as a broader biomarker of health. Bohannon [15] demonstrated that handgrip force is consistently associated with overall strength, upper-limb function, bone mineral density, falls, multimorbidity, and quality of life, reinforcing its translational relevance. Clinically, grip strength has also been shown to serve as a simple prognostic indicator of nutritional status and surgical outcomes [16]. Advances in multi-sensor-based instrumentation now provide the precision needed to detect subtle asymmetries in load distribution, allowing integration of sport-performance assessment with clinical monitoring [13,17].

Building on these developments, force-sensitive resistors (FSRs) offer a practical and nonintrusive solution for quantifying finger-specific forces under sport-relevant conditions. They can be embedded into fencing handles without altering external geometry, enabling detailed mapping of individual finger contributions to total grip force [18,19]. FSR-type or other thin-film pressure-sensor systems have already been implemented in sport-specific equipment—including tennis rackets, climbing holds, and golf clubs—to visualize dynamic pressure patterns and identify muscle activation strategies associated with fatigue or improper technique [11,12,14]. Such instrumentation offers the potential not only to refine performance analysis but also to monitor rehabilitation progress and detect maladaptive load-sharing strategies that may predispose athletes to injury.

Therefore, this pilot study aimed to test a novel FSR-instrumented fencing handle for quantifying finger-specific grip strength and coordination, with a focus on identifying whether individual load-sharing patterns can be reliably captured in a way that may support clinical assessment, rehabilitation monitoring, and injury prevention in fencing athletes. A deeper understanding of grip control mechanisms in this sport-specific context may also inform broader clinical applications, including targeted rehabilitation strategies and the ergonomic design of hand-held implements.

## 2. Materials and Methods

### 2.1. Participants

Eight male épée fencers were recruited for this pilot study. All were active members of the Polish national team and reported more than 10 years of competitive training experience. Inclusion criteria required regular participation in training and competition, absence of any upper-limb injury in the preceding six months, and the use of each athlete’s own weapon and preferred grip type during testing. Detailed characteristics of fencers were provided in Table 1.

The study was conducted in accordance with the principles outlined in the Declaration of Helsinki. The University Bioethics Committee approved it for Scientific Research at the Academy of Physical Education in Katowice (Resolution No. 7-VI/2025, dated 5 June 2025). Written informed consent was obtained from all participants before data collection.

### 2.2. Instrumentation

Maximal isometric handgrip force was assessed using a standard hydraulic hand dynamometer (Model SH5001, SAEHAN Corp., Changwon, Republic of Korea), which provided a global reference measure of maximal grip force. This approach has long been validated for clinical assessment, with studies confirming strong test–retest reliability and construct validity for grip and pinch measurements [20].

**Figure 1:**
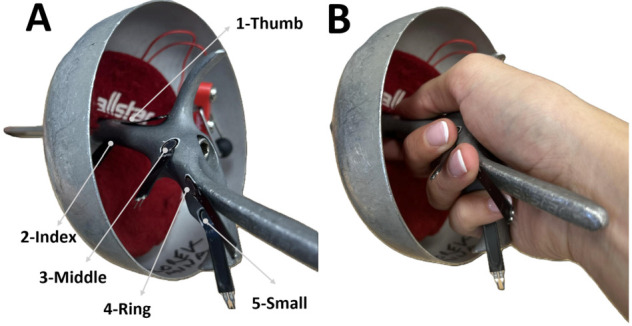
(**A**) Location of the five force-sensitive resistors (FSRs) embedded in the anatomical handle for right-handed fencers: thumb (1), index (2), middle (3), ring (4), little finger (5). (**B**) Standardized digital positioning on the pistol grip handle of the fencing épée.

Grip pressure on the fencing weapon was then recorded using five ultra-thin force-sensitive resistors (FSRs) (model CZN-CP1, FSR-400, IEE, Luxembourg) with a measurement range of 0.2–100 N, an operating current of 1 mA, and an operating temperature range of −40 °C to +75 °C. Each sensor had a circular sensing area with a diameter of 7.62 mm and a thickness of 0.34 mm. The small footprint and minimal thickness allowed integration into an anatomically contoured épée grip without altering its external dimensions or tactile characteristics. FSR sensors were mounted on each participant’s own weapon immediately before testing, with placement verified to ensure each sensor lay beneath the corresponding finger (Figure 1). The anatomically shaped handle is designed symmetrically for right- and left-handed fencers—its structure remains identical, differing only in mirrored orientation to match the fencer’s dominant hand.

Each FSR was wired to a custom-made connector board that grouped the sensor outputs and supplied the reference circuitry. The connector board was linked to a Kistler data acquisition unit (Kistler, Winterthur, Switzerland), enabling all channels to be recorded using BioWare software (version 5.3.0, Kistler, Switzerland). A regulated DC power supply was set to 10 V to ensure a stable excitation voltage for the sensors.

Before testing, the sensors were calibrated against a Kistler force platform (Kistler, Switzerland). During calibration, the sensors were mounted on the platform surface and subjected to a series of incremental loads, which enabled the derivation of voltage-to-force conversion curves for subsequent data transformation. The calibration procedure yielded a coefficient of determination (R^2^ = 0.90), indicating good correspondence between sensor output and reference forces.

### 2.3. Procedures

Handgrip strength was first assessed with a standard handheld dynamometer [21]. Participants were seated with the testing arm fully extended alongside the trunk and the wrist in a neutral position. Each participant performed two maximal squeezes with the dominant hand, separated by a 60-s rest interval, and the higher of the two values displayed by the device was recorded as maximal grip strength (kgf).

Subsequently, participants completed task-specific measurements with the instrumented épée handle. They were seated in a standardized position, with the forearm unsupported and the blade held freely to allow natural stabilization of the weapon without extraneous support on the testing surface. The maximal squeeze protocol consisted of two 5-s maximal voluntary isometric contractions (MVCs) for each of the three hand configurations, which were presented in randomized order:

- Pinch—radial digital grip (thumb–index opposition): the hand grasped the handle with all fingers, but participants were instructed to exert maximal force specifically with the index finger and thumb.
- Trio—ulnar digital flexion task (middle, ring, small fingers): the hand grasped the handle with all fingers, but participants were instructed to contract the middle, ring, and small fingers maximally.
- Whole—composite digital grasp (all five digits): participants were instructed to maximally contract with all fingers simultaneously.

In all conditions, the weapon was held in a natural fencing grip with all fingers in contact to ensure consistent positioning. To minimize unintended compensations and allow participants to focus on the instructed finger group, the blade was lightly supported with the non-dominant hand throughout testing. This stabilization ensured that grip forces were generated primarily by the designated fingers while maintaining overall weapon stability. Trials for a given configuration were grouped; between the two 5-s MVC trials, the participant rested for 60 s to minimize fatigue. Participants were instructed to produce maximal force “as fast and as hard as possible” at the start of each trial and to maintain the effort for the full 5 s. No visual feedback of output was provided during MVCs; participants relied on proprioceptive and tactile cues.

### 2.4. Data Processing

All analyses were performed in MATLAB R2024a (MathWorks, Carlsbad, CA, USA). Raw voltage signals from each FSR channel were visually screened for artifacts. Signals were sampled at 1000 Hz and pre-processed with a 5-point median filter to suppress impulsive noise. They were then low-pass filtered using a 4th-order zero-phase Butterworth filter with a 5 Hz cutoff to reduce high-frequency noise and preserve the dynamic profile of voluntary grip forces, following established approaches in hand and finger-force analysis using strain-gauge and FSR-type sensors [22,23,24]. Voltages were converted to force using calibration curves derived from the Kistler force platform.

For each 5-s MVC trial, a moving mean (window = 2.0 s) was computed; the highest value of the smoothed trace within the bout was taken as the plateau mean. Baseline force was estimated from the 1-s period immediately preceding the start of each MVC, and the final MVC value was expressed as the absolute difference between plateau and baseline (|plateau − baseline|). When two MVC trials were available for a given configuration, the larger baseline-corrected value was retained. All primary outcomes are therefore reported as baseline-corrected plateau means (N). To derive functional grip configurations, forces from individual sensors were combined as follows:

- Pinch—the sum of the index finger and thumb sensors
- Trio—sum of middle, ring, and small finger sensors
- Whole—sum of all five finger sensors

Thus, cross-task ratios (Pinch/Whole and Trio/Whole) were calculated by comparing MVC values obtained in separate Pinch, Trio, and Whole trials. Within-Whole ratios (Pinch-in-Whole and Trio-in-Whole) were calculated as the relative contributions of these subgroups during the Whole trial itself.

### 2.5. Statistics

Descriptive statistics (means ± standard deviations) were calculated for maximal grip forces (N) obtained with the handgrip dynamometer and the instrumented handle (Whole-FSR), for maximal forces in Pinch, Trio, and Whole conditions, for cross-task ratios (Pinch/Whole, Trio/Whole), for within-Whole shares (Pinch-in-Whole, Trio-in-Whole), and for individual finger contributions during the Whole condition. Data normality was verified using the Shapiro–Wilk test and confirmed for all variables; however, given the small sample size (n = 8) and to minimize potential bias from non-normal distributions or outliers, the non-parametric Wilcoxon signed-rank test for dependent measures was applied to compare force values (N) between cross-task Pinch and Pinch-in-Whole, as well as between cross-task Trio and Trio-in-Whole (results shown as median ± interquartile range). All analyses were performed using Statistica software v.13.3 (TIBCO Software Inc., Santa Clara, CA, USA).

### 2.6. Use of GenAI

During the preparation of this work, the authors used ChatGPT (GPT-5, OpenAI) only to improve the clarity and readability of the text. After using this tool, the authors reviewed and edited all content as needed and take full responsibility for the final version of the manuscript.

## 3. Results

### 3.1. Participant Characteristics

Eight male épée fencers took part in the study (Table 1). The athletes were, on average, 23.6 ± 5.0 years old, with a mean height of 185.1 ± 5.2 cm and a body mass of 81.3 ± 7.7 kg. Handedness distribution included seven right-handed and one left-handed participant. Conventional handgrip dynamometry yielded maximal strength values of 65.3 ± 11.6 kgf (≈640 ± 114 N), with individual results ranging from 45 to 81 kgf.

### 3.2. Comparison of Handgrip Strength by Dynamometer and Instrumented Handle (Whole-FSR)

Whole-hand MVCs recorded with the instrumented handle represented on average 22.8 ± 9.2% of maximal forces obtained with conventional handgrip dynamometry, with individual values ranging from 11.1% to 40.5% (Table 2).

### 3.3. Finger-Group Contributions to Maximum Grip Strength (Pinch, Trio, Whole-Hand)

Whole-hand output varied substantially across participants (70.9–226.6 N) (Table 3). In isolated tasks, maximal force was higher for the Pinch subgroup (87.9 ± 12.0 N) than for the Trio (70.4 ± 18.3 N), yielding cross-task Pinch/Whole and Trio/Whole ratios of 69.0 ± 24.8% and 55.5 ± 25.6%, respectively. These ratios reflect performance in separate trials, and in some athletes, Pinch or Trio exceeded 100% of Whole (e.g., P6, P8). This indicates that, when tested in isolation, specific finger subgroups were capable of producing higher force than they contributed during the integrated Whole-hand condition. By contrast, within the Whole-hand condition itself, the relative distribution of forces was ulnar-biased, with Pinch-in-Whole contributing 38.8 ± 9.9% and Trio-in-Whole 61.2 ± 9.9%. This within-task pattern was also heterogeneous: some fencers showed radial (Pinch) dominance (e.g., P2), whereas others displayed stronger ulnar-side (Trio) involvement (e.g., P5).

**Figure 2:**
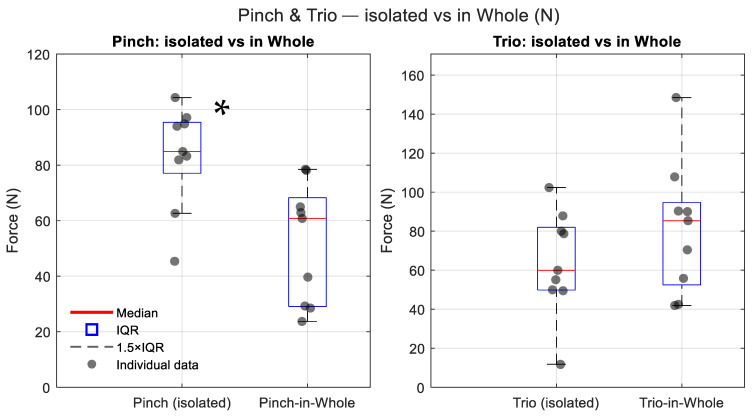
Box plots comparing isolated Pinch and Trio forces with their integrated contributions during Whole-hand MVCs. Legend: IQR—Interquartile Range. * indicates a statistically significant difference (*p* < 0.05).

Direct comparison of isolated and integrated finger forces revealed significant differences for the Pinch configuration (Figure 2). Pinch MVCs recorded in isolation (median 83.0 ± 29.2 N) were significantly higher than Pinch-in-Whole contributions (median 54.7 ± 16.3 N); Wilcoxon signed-rank test, Z = 2.52, *p* = 0.012). By contrast, Trio forces measured in isolation (66.3 ± 24.5 N) did not differ significantly from their integrated contribution during Whole-hand MVCs (median 86.3 ± 24.6 N, Z = 1.12, *p* = 0.263).

### 3.4. Finger Contributions During the Whole-Hand Maximum Strength Task

**Figure 3:**
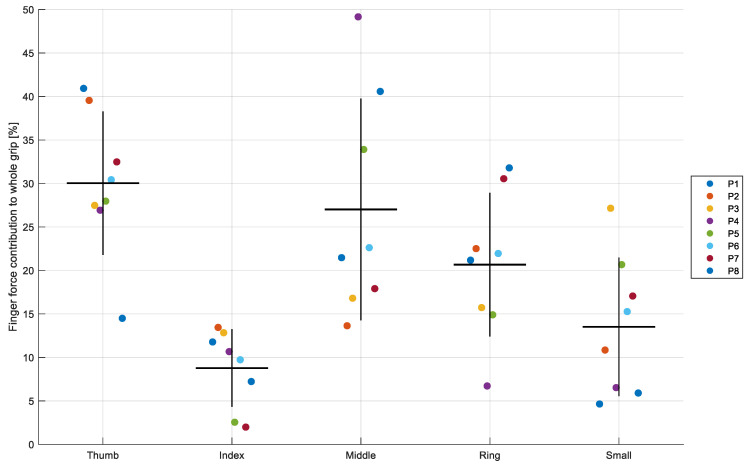
Percentage contribution of each finger to the total grip force across participants during the Whole-hand condition. Each dot represents the relative contribution (% of total grip force) for an individual participant (P1–P8), with colors indicating participants. Black horizontal lines show group means, and vertical bars indicate standard deviation (SD).

Figure 3 summarizes the distribution of finger-specific maximal forces across the eight épée fencers. On average, the thumb generated the highest force output (≈30 N), followed by the middle (≈27 N) and ring fingers (≈21 N). The index finger contributed more modestly (≈9 N), while the small finger showed the lowest average force (≈13 N). This gradient was consistent across most athletes, although notable inter-individual variability was observed.

For example, P4 produced an exceptionally high middle-finger output (49 N), nearly double the group mean, whereas P5 relied more on the small finger (21 N) than most of his peers. In contrast, P7 displayed a relatively balanced distribution between the ring and thumb, both exceeding 30 N. Despite these outliers, the overall pattern indicates a dominant role of the thumb and middle finger in generating maximal grip force, with the index and small fingers contributing substantially less.

## 4. Discussion

This pilot study aimed to evaluate a novel FSR-instrumented épée handle for quantifying finger-specific grip strength and coordination in elite fencers. The findings demonstrate that the system was able to capture detailed finger-level load distribution during maximal voluntary contractions, revealing both group-level patterns and meaningful inter-individual variability. Significantly, the device differentiated between Pinch, Trio, and Whole-hand contributions, highlighting its potential to complement standard dynamometry by providing a more granular, sport-specific assessment of grip function.

In this study, maximal grip strength recorded with a standard dynamometer (65.3 ± 11.7 kgf) was substantially higher than the sum of finger forces in the Whole-hand condition measured with the instrumented handle (141.0 ± 43.4 N ≈ 14.4 ± 4.4 kgf). This discrepancy reflects methodological differences: dynamometry integrates the combined force of multiple fingers and wrist support, whereas the FSRs capture only the pressure transmitted through each finger–handle contact point. Clinically, this resolution is highly relevant. Global dynamometry may mask selective weakness or under-recruitment of individual fingers, even when total force appears normal. Such hidden imbalances could predispose athletes to overuse syndromes or compensatory strategies, for example, overloading the thumb–index pair. As emphasized by Sasaki et al. [24], conventional grip dynamometers cannot capture the temporal evolution or coordination of finger motion, limiting their diagnostic resolution. Their study in carpal tunnel syndrome patients demonstrated that the disorder affects not only maximal strength but also the fine coordination of finger forces, highlighting the value of finger-specific monitoring. By extension, in fencing, detecting disproportionate reliance on radial (index and thumb) versus ulnar digits (middle, ring, small) could guide individualized rehabilitation, finger-specific strengthening, or ergonomic adjustments of the handle to reduce localized stress and injury risk.

Cross-task ratios comparing Pinch and Trio against Whole-hand MVCs revealed marked variability across athletes. On average, the Pinch/Whole ratio reached 69.0 ± 24.8%, whereas the Trio/Whole ratio was 55.5 ± 25.6%. In some fencers, Pinch forces even exceeded 100% of the Whole condition (e.g., P6, 115%), suggesting that isolated index–thumb contractions can, under certain conditions, outperform their integrated contribution within the full grip. This finding aligns with biomechanical studies of pinch grips, which demonstrate that the thumb–index complex is capable of generating disproportionately high forces in isolation and is often prioritized in precision tasks that require stability and control [23]. Clinically, such Pinch-dominant patterns may reflect compensatory recruitment strategies, potentially overload the carpometacarpal joint of the thumb and increasing susceptibility to overuse injury.

Beyond sport-specific considerations, the observed discrepancies between isolated and integrated finger forces can also be interpreted in the context of established neurophysiological constraints on multi-digit coordination. One well-documented phenomenon is enslaving, defined as the involuntary activation of fingers that are not directly involved in a task [25]. Enslaving reflects both anatomical coupling and shared neural drive, and it has been shown to vary across populations, being accentuated in stroke patients [26] and altered in aging or Parkinson’s disease [27,28,29]. A related constraint is the force deficit, where the total force generated during simultaneous activation of all fingers is lower than the summed capacity of the same digits tested individually [30]. These mechanisms highlight the limits of finger independence and coordination, and may partly explain why the index–thumb pair in our study produced higher outputs in isolation than within the integrated Whole-hand grip. As reviewed by Schieber and Santello [31], such neuromuscular interactions arise from short-term synchronization of motor units and co-contraction across synergistic muscles, further complicating the interpretation of load-sharing patterns. Taken together, these neurophysiological principles provide a broader explanatory framework for our findings and emphasize the need for integrative approaches, ideally combining force-sensing technology with EMG, to fully characterize hand function in both sport and clinical populations. In summary, these neuromuscular mechanisms—enslaving, force deficit, and inter-digit coupling—collectively explain the observed distribution of grip forces and underline the importance of multi-digit coordination in precision tasks such as fencing.

Within-Whole ratios, which quantify the relative contribution of finger groups during the Whole-hand MVC, revealed a distribution distinct from cross-task ratios. On average, the Pinch subgroup (thumb + index) accounted for 38.8 ± 9.9% of Whole output, whereas the Trio subgroup (middle, ring, small) accounted for 61.2 ± 9.9%. Importantly, isolated Pinch MVCs (median 83.0 ± 29.2 N) were significantly higher than their integrated Pinch-in-Whole contributions (median 54.7 ± 16.3 N; Wilcoxon signed-rank test, Z = 2.52, *p* = 0.012), yielding a lower Pinch/Whole ratio (mean 69.0 ± 24.8%) compared to the within-Whole estimate. By contrast, Trio forces in isolation (median 66.3 ± 24.5 N) did not differ significantly from their integrated contribution (median 86.3 ± 24.6 N; Z = 1.12, *p* = 0.263). This asymmetry indicates that the index–thumb pair produces less force when integrated into the Whole grip, whereas the ulnar Trio maintains its performance across contexts. Functionally, this pattern may reflect a neuromuscular coordination tendency in which radial digits downscale their output during combined gripping, allowing the ulnar side to sustain load and contribute to overall grip stability.

Such a shift is consistent with multi-finger coordination studies, which show that ulnar digits contribute disproportionately to grip stability and sustained force. In contrast, radial digits specialize in precision and fine force control [22]. Experimental studies using selective finger engagement on dynamometers have shown that removal of ulnar digits disproportionately reduces total grip force, confirming their stabilizing role within the synergy [32]. While such models are simplified compared with fencing-specific grips, they illustrate general patterns of differentiated finger contribution to total force generation. In our data, similar asymmetries were reflected by discrepancies between isolated Pinch and integrated Whole performance, suggesting that these differences arise not only from mechanical strength distribution but also from neuromuscular coordination demands.

Finger-level analysis in this study revealed distinct patterns of load distribution across digits. On average, the thumb generated the highest forces (30.4 ± 8.4 N), followed by the middle (27.2 ± 10.1 N), ring (21.0 ± 7.2 N), small (13.2 ± 6.4 N), and index finger (9.4 ± 4.0 N). This hierarchy deviates from conventional reference patterns in healthy adults, where the middle finger typically dominates (~30–35%) and the index finger contributes more substantially [32,33]. In fencers, however, the thumb’s output was comparatively greater, while the index played only a minor role.

At the individual level, several atypical strategies were observed. P4 produced exceptionally high middle-finger force (49 N), whereas P5 showed a substantial reliance on the small finger (21 N). P7 displayed balanced high outputs of both thumb and ring (>30 N), suggesting a more distributed strategy. Such heterogeneity indicates that athletes achieve effective grip through different neuromuscular solutions rather than a fixed template.

From a clinical perspective, these deviations may carry relevance for injury surveillance and rehabilitation. Excessive thumb loading could predispose athletes to carpometacarpal joint stress, while consistently low index contributions may indicate inefficient radial-side force transfer. Conversely, stronger engagement of the small finger, as seen in some athletes, might reflect adaptive recruitment of ulnar support but could also increase the risk of localized fatigue. Comparable clinical evidence shows that reduced grip and pinch strengths correlate negatively with indices of rheumatoid arthritis activity and disability [34], and similar monitoring approaches have been emphasized in osteoarthritis patients, where grip and pinch strength provide reproducible and clinically relevant feedback [35]. Identifying such patterns in athletes could therefore guide individualized conditioning, targeted therapeutic exercise, or ergonomic refinements of weapon handles.

From a performance standpoint, such finger-resolved load data could also provide actionable feedback for coaches and performance specialists. Tracking individual grip patterns over time may help identify early signs of fatigue or asymmetrical loading, supporting the optimization of training load and the prevention of overuse injuries. Moreover, this approach could complement return-to-play criteria after wrist or finger pathologies by providing quantitative evidence of functional recovery and load rebalancing.

Comparable approaches have been applied in racket sports and occupational ergonomics, where pressure-sensing systems and instrumented handles are used to monitor hand–equipment interaction. In tennis and golf, for example, FSR-type sensors have revealed sport-specific pressure signatures that correlate with technical efficiency and forearm muscle activation patterns [11,36]. Similarly, climbing studies have used multi-sensor arrays to detect asymmetric loading and fatigue-related adaptations [13]. These parallels highlight the broader applicability of finger-resolved force sensing across domains, bridging performance monitoring, injury prevention, and rehabilitation.

### 4.1. Limitations

This pilot study has several limitations that should be acknowledged. First, although the sample size was limited (n = 8), it consisted of elite male fencers, which represents an informative cohort for preliminary validation at the highest performance level. Nevertheless, this may limit generalizability to other levels of expertise, female athletes, or youth populations.

Second, the study focused exclusively on maximal voluntary contractions, whereas fencing performance relies heavily on dynamic, submaximal, and fatigued states that were not examined here.

Third, although the instrumented handle captured finger-specific forces, it did not measure muscle activity directly (e.g., via EMG) or account for kinetic contributions from the wrist and forearm, which likely interact with digital load sharing. Nevertheless, this resolution remains clinically valuable, as global dynamometry may mask selective weakness or under-recruitment of individual fingers, even when total force distribution profiles appear normal.

Fourth, the sensing elements had a relatively small active diameter (7.62 mm). While sensor placement was carefully standardized and monitored, subtle finger repositioning or slipping during forceful squeezes may have led to partial off-loading of the sensor surface, potentially underestimating true force in some trials. Additionally, the curvature mismatch between the flat FSR surface and the contoured handle could slightly reduce contact uniformity under high loads, leading to minor underestimation of actual finger forces.

Finally, inter-session reliability was not assessed, as all participants completed a single experimental session. Although the thumb was included in the Whole-hand condition, its dynamic contribution to grip modulation and weapon control was not specifically analyzed in this study and warrants targeted investigation in future experiments.

### 4.2. Future Directions

Future work should expand this methodology to larger and more diverse cohorts, including female and junior fencers, to establish normative reference data and sport-specific adaptations. Including athletes with different technical proficiency levels could also help determine whether skill-related factors influence the correspondence between dynamometer and instrumented-handle measures. Longitudinal studies are necessary to investigate whether finger load-sharing profiles evolve with training, fatigue, or rehabilitation. Combining the FSR-instrumented handle with surface electromyography, motion capture, or pressure-sensitive gloves could provide a more complete picture of the neuromechanical strategies underlying grip control. Importantly, prospective studies should test whether abnormal or asymmetric finger-force distributions predict the onset of overuse symptoms in the hand, wrist, or forearm, thereby validating the tool’s clinical utility for injury prevention and return-to-play monitoring. Finally, future experiments should incorporate more ecologically valid tasks, ideally including fencing actions or even competitive bouts, to evaluate how finger-force coordination manifests under realistic combat conditions. Such time-resolved analyses will be essential to capture how digital tension increases or decreases throughout technical execution and how these coordination patterns differ between individual fencers. Ergonomic refinements of handle design based on finger-force mapping could then help optimize both performance and athlete safety.

## 5. Conclusions

This pilot study demonstrates the feasibility of using an FSR-instrumented épée handle to capture finger-specific grip contributions in elite fencers. By complementing standard dynamometry with sport-specific, digit-level resolution, this method shows translational potential for detecting maladaptive grip strategies and guiding rehabilitation and injury-prevention programs. The system successfully differentiated between Pinch, Trio, and Whole-hand contributions, capturing both group-level tendencies and substantial inter-individual variability. Compared with standard dynamometry, which provides only a global measure of grip strength, the instrumented handle revealed finger-specific load-sharing strategies that may have clinical and performance relevance. The results highlight the dominant contribution of the thumb and middle finger, the stabilizing role of ulnar digits, and athlete-specific neuromuscular adaptations that could inform individualized conditioning, rehabilitation, or ergonomic refinements of handle design. These findings establish a proof of concept for finger-level assessment in fencing and support future studies that extend the method to dynamic, ecologically valid tasks and longitudinal monitoring of injury risk.

## Figures and Tables

**Table 1:** Participant characteristics. Age, height, body mass, handgrip strength, and handedness of the eight épée fencers.

Participant ID	Age (Years)	Height (cm)	Mass (kg)	Grip Strength (kgf)	Handness
P1	20	183	80	68	R
P2	31	185	98	81	R
P3	28	190	88	74	R
P4	31	182	80	75	R
P5	18	174	71	45	L
P6	20	178	81	65	R
P7	19	181	75	57	R
P8	22	188	82	57	R
Mean ± SD	23.6 ± 5.0	185.1 ± 5.2	81.3 ± 7.7	65.3 ± 11.6	87.5% R

Legend: Values of grip strength are expressed in kgf (mean ± SD also reported); 1 kgf ≈ 9.81 N. Handedness: R—right-handed and L—left-handed.

**Table 2:** Comparison between conventional handgrip dynamometer values and Whole-hand maximum voluntary contractions (MVCs) measured with the instrumented handle.

ID	Grip Dynamometer (N)	Whole-FSR (N)	Ratio FSR/Dyn (%)
P1	667.1	148.91	22.3
P2	794.6	118.71	14.9
P3	726	150.82	20.8
P4	735.8	172.8	23.5
P5	441.5	129.99	29.4
P6	637.7	70.97	11.1
P7	559.2	226.62	40.5
P8	559.2	109.03	19.5
Mean ± SD	640 ± 114	141 ± 43	22.8 ± 9.2

Legend: Grip strength expressed in Newtons (N). Ratio calculated as Whole-FSR ÷ dynamometer × 100%. Values are presented individually for each participant and as the group mean ± SD.

**Table 3:** Maximal grip strength (N) and finger-group contributions in eight fencers.

ID	Pinch (N)	Trio (N)	Whole (N)	Pinch/Whole (%)	Trio/Whole (%)	Pinch-in-Whole (%)	Trio-in-Whole (%)
P1	94.87	59.95	148.91	63.71	40.26	52.71	47.29
P2	97.14	49.45	118.71	81.83	41.65	52.99	47.01
P3	84.90	78.69	150.82	56.29	52.18	40.31	59.69
P4	94.01	49.93	172.80	54.40	28.89	37.60	62.40
P5	62.64	102.34	129.99	48.19	78.73	30.52	69.48
P6	81.89	80.00	70.97	115.39	112.71	40.16	59.84
P7	83.25	87.84	226.62	36.74	38.76	34.47	65.53
P8	104.36	55.10	109.03	95.71	50.54	21.72	78.28
M	87.88	70.41	140.98	69.03	55.47	38.81	61.19
SD	11.97	18.33	43.37	24.76	25.64	9.87	9.87

Note: Cross-task ratios express the capacity of Pinch or Trio groups relative to the Whole total when tested separately (may exceed 100%), whereas within-Whole shares denote the actual allocation adopted when all five fingers act together (sum to 100%). The two measures, therefore, need not coincide. Legend: M—mean, and SD—standard deviation.

## Data Availability

Template data collection forms, extracted data from included studies, datasets used in all analyses can be provided by the corresponding author upon reasonable request.

## References

[1] Chen T.L.-W., Wong D.W.-C., Wang Y., Ren S., Yan F., Zhang M. (2017). Biomechanics of Fencing Sport: A Scoping Review. PLoS ONE.

[2] Chang C.L., Li K.W., Lin F.T., Jou Y.T., Huang C. (2009). Der A Study of Optimal Handle Shape and Muscle Strength Distribution on Lower Arm When Holding a Foil. Percept. Mot. Ski..

[3] Lin F.L., Chang C.L., Jou Y.T., Pan H.C., Hsu T.Y. The Study of Influence of Fencing Handle Type and Handle Angle on Wrist for a Fencing Game. Proceedings of the 2010 IEEE 17th International Conference on Industrial Engineering and Engineering Management, IE and EM2010.

[4] Kai W.L., Chih-Lin C. Effects of Handwear and Hand Posture on Four Performance Measures in Foil Fencing. Proceedings of the Human Factors and Ergonomics Society Annual Meeting.

[5] Witkowski M., Bojkowski Ł., Karpowicz K., Konieczny M., Bronikowski M., Tomczak M. (2020). Effectiveness and Durability of Transfer Training in Fencing. Int. J. Environ. Res. Public Health.

[6] Tsolakis C., Kostaki E., Vagenas G. (2010). Anthropometric, Flexibility, Strength-Power, and Sport-Specific Correlates in Elite Fencing. Percept. Mot. Ski..

[7] Dresp-Langley B., Nageotte F., Zanne P., de Mathelin M. (2020). Correlating Grip Force Signals from Multiple Sensors Highlights Prehensile Control Strategies in a Complex Task-User System. Bioengineering.

[8] Harmer P.A. (2008). Incidence and Characteristics of Time-Loss Injuries in Competitive Fencing: A Prospective, 5-Year Study of National Competitions. Clin. J. Sport Med..

[9] Swatowska M.M., Akbaş A., Juras G. (2020). Injuries in High-Performance Fencers—A Review. Arch. Budo.

[10] Stanicki B., Criscione J.X., Shaari A.L., Thompson K., Galdi B. (2025). An Analysis of Fencing Injuries in the United States: A 10-Year Database Review. Orthop. J. Sports Med..

[11] Komi E.R., Roberts J.R., Rothberg S.J. (2008). Measurement and Analysis of Grip Force during a Golf Shot. Proc. Inst. Mech. Eng. Part P J. Sports Eng. Technol..

[12] Irie K., Yokota J., Takeda M., Mukaiyama K., Nishida Y., Sato M., Mishima Y., Yamamoto N., Nagai-Tanima M., Aoyama T. (2022). Comparison of Forearm Muscle Activation and Relationship with Pressure Distribution in Various Grip Patterns. Asian J. Occup. Ther..

[13] Fuss F.K., Niegl G. (2006). Instrumented Climbing Holds and Dynamics of Sport Climbing. Eng. Sport 6.

[14] Zhang K., Guo B., Yang M., Jia Y., Zhang K., Wang L. (2024). The Assessment of Sports Performance by Grip Pressure Using Flexible Piezoresistive Pressure Sensors in Seven Sports Events. Sci. Rep..

[15] Bohannon R.W. (2019). Grip Strength: An Indispensable Biomarker for Older Adults. Clin. Interv. Aging.

[16] Hunt D.R., Rowlands B.J., Johnston D. (1985). Hand Grip Strength—A Simple Prognostic Indicator in Surgical Patients. J. Parenter. Enter. Nutr..

[17] Esser S., Taylor W.C., Bertasi R.A.O., Nishi L., Heckman M.G., Abadin A., Vomer R., Pujalte G.G.A. (2024). Effects of Grip Style and Contact Point on Force Production in a Tennis Forehand Groundstroke. Cureus.

[18] Tornifoglio S.V., Peterson D.R. Determining the Minimum Number of Thin-Film Force Sensors Required to Represent Actual Hand Grip Forces. Proceedings of the 2012 38th Annual Northeast Bioengineering Conference (NEBEC).

[19] Watanabe K., Kinoshita H., Ishido W., Hashiguchi T. (2024). Racket Holding Forces for Playing A Forehand Stroke at Varied Swing Speeds in Table Tennis. ISBS Proc. Arch..

[20] Mathiowetz V., Weber K., Volland G., Kashman N. (1984). Reliability and Validity of Grip and Pinch Strength Evaluations. J. Hand Surg. Am..

[21] Roberts H.C., Denison H.J., Martin H.J., Patel H.P., Syddall H., Cooper C., Sayer A.A. (2011). A Review of the Measurement of Grip Strength in Clinical and Epidemiological Studies: Towards a Standardised Approach. Age Ageing.

[22] Li Z.M., Zatsiorsky V.M., Latash M.L. (2000). Contribution of the Extrinsic and Intrinsic Hand Muscles to the Moments in Finger Joints. Clin. Biomech..

[23] Valero-Cuevas F.J., Zajac F.E., Burgar C.G. (1998). Large Index-Fingertip Forces Are Produced by Subject-Independent Patterns of Muscle Excitation. J. Biomech..

[24] Sasaki T., Makino K., Nimura A., Suzuki S., Kuroiwa T., Koyama T., Okawa A., Terada H., Fujita K. (2020). Assessment of Grip-Motion Characteristics in Carpal Tunnel Syndrome Patients Using a Novel Finger Grip Dynamometer System. J. Orthop. Surg. Res..

[25] Kilbreath S.L., Gandevia S.C. (1994). Limited Independent Flexion of the Thumb and Fingers in Human Subjects. J. Physiol..

[26] Lang C.E., Schieber M.H. (2004). Human Finger Independence: Limitations Due to Passive Mechanical Coupling versus Active Neuromuscular Control. J. Neurophysiol..

[27] Mirakhorlo M., Van Beek N., Wesseling M., Maas H., Veeger H.E.J., Jonkers I. (2018). A Musculoskeletal Model of the Hand and Wrist: Model Definition and Evaluation. Comput. Methods Biomech. Biomed. Eng..

[28] Gelb D.J., Oliver E., Gilman S. (1999). Diagnostic Criteria for Parkinson Disease. Arch. Neurol..

[29] Park D.C., Lodi-Smith J., Drew L., Haber S., Hebrank A., Bischof G.N., Aamodt W. (2014). The Impact of Sustained Engagement on Cognitive Function in Older Adults: The Synapse Project. Psychol. Sci..

[30] Ohtsuki T. (1981). Inhibition of Individual Fingers during Grip Strength Exertion. Ergonomics.

[31] Schieber M.H., Santello M. (2004). Hand Function: Peripheral and Central Constraints on Performance. J. Appl. Physiol..

[32] Talsania J.S., Kozin S.H. (1998). Normal Digital Contribution to Grip Strength Assessed by a Computerized Digital Dynamometer. J. Hand Surg. Br..

[33] Cha S.M., Shin H.D., Kim K.C., Park J.W. (2014). Comparison of Grip Strength among 6 Grip Methods. J. Hand Surg. Am..

[34] Dedeoǧlu M., Gafuroǧlu Ü., Yilmaz Ö., Bodur H. (2013). The Relationship Between Hand Grip and Pinch Strengths and Disease Activity, Articular Damage, Pain, and Disability in Patients with Rheumatoid Arthritis. Arch. Rheumatol..

[35] Ziv E., Patish H., Dvir Z. (2008). Grip and Pinch Strength in Healthy Subjects and Patients with Primary Osteoarthritis of the Hand: A Reproducibility Study. Open Orthop. J..

[36] Mukaiyama K., Irie K., Takeda M., Yamashita R., Uemura S., Kanazawa S., Nagai-Tanima M., Aoyama T. (2022). Load Distribution and Forearm Muscle Activity during Cylinder Grip at Various Grip Strength Values. Hand Surg. Rehabil..

